# Electrical control of Förster resonant energy transfer across single-layer graphene

**DOI:** 10.1515/nanoph-2021-0778

**Published:** 2022-06-10

**Authors:** Yansheng Liu, Miguel Angel Niño Ortí, Feng Luo, Reinhold Wannemacher

**Affiliations:** IMDEA Nanoscience, calle Faraday 9, Ciudad Universitaria de Cantoblanco, 28049, Madrid, Spain; School of Science, Universidad Autónoma de Madrid, Ciudad Universitaria de Cantoblanco, 28049, Madrid, Spain; Guangxi University of Science and Technology, School of Electrical and Information Engineering, 268 Donghuan Avenue, 545006 Liuzhou City, Guangxi Province, People’s Republic of China

**Keywords:** Förster resonant energy transfer, fluorescence, non-local optics, single layer graphene

## Abstract

In artificial structures of molecular or quantum dot emitters in contact with single-layer graphene (SLG) Förster-type resonant energy transfer (FRET) can occur unconditionally due to the gapless band structure of SLG. A significant breakthrough for applications, however, would be the electrical modulation of FRET between arbitrary FRET pairs, using the SLG to control this process and taking advantage of the particular band structure and the monatomic thickness of SLG, far below the typical Förster radius of a few nanometers. For a proof of concept, we have therefore designed a Sandwich device where the SLG was transferred onto holey Si_3_N_4_ membranes and organic molecules were deposited on either side of the SLG. The relative photoluminescence (PL) intensities of donor and acceptor molecules changed continuously and reversibly with the external bias voltage, and a variation of about 6% of FRET efficiency has been achieved. We ascribe the origin of the electrical modulation of FRET to important doping-dependent nonlocal optical effects in the near field of SLG in the visible range.

## 1 Introduction

FRET [1], the nonradiative transfer of energy between a donor and an acceptor by long-range dipole–dipole interactions, has found many practical applications for over 60 years. FRET plays a central role in many domains such as biosensors [2, 3], real-time monitoring systems for drug release [4], fluorescent probes [5], DNA detection [6], solar energy conversion [7], or optoelectronic devices [8]. In this process, the essential condition is that the acceptor can absorb the energy at the emission wavelength of the donor [9, 10]. The rate of energy transfer can be affected by many factors, for instance, the extent of spectral overlap, the relative orientation of the transition dipoles, and the distance between the donor and acceptor molecules [2]. When FRET happens, the energy transfer from the donor to the acceptor can be measured with low background interference. This has been applied for many analytical purposes, in particular in fluorescence microscopy [11, 12].

Recently, there is a growing interest in the FRET process between emitters and 2-dimensional (2D) materials, in particular, graphene [13], [14], [15], [16], [17], [18]. Single-layer graphene (SLG) as a two-dimensional (2D) structure of carbon atoms possesses many unusual properties and has attracted the attention of many researchers since the first report in the literature [19]. It consists of sp^2^-hybridized carbon which determines its unique honeycomb lattice structure [20]. SLG is considered as a zero-gap semimetal with zero overlap between its valence and conduction bands [19, 21]. This makes graphene a very good energy acceptor for various kinds of donors. In addition, graphene possesses some other unique properties, such as high absorbance of light with negligible dependence on the wavelength, excellent thermal and chemical stability, high conductivity, and large specific surface area [22], [23], [24]. According to these unique and outstanding properties, SLG has been considered as a promising candidate for the next-generation material in optical and optoelectronic applications. Benefitting from these unique properties many kinds of devices and hybrid systems have been designed with new functionalities and enhanced optoelectronic properties. The FRET process between emitters and graphene was studied mainly focusing on the energy transfer from QDs to graphene [17, 25], [26], [27], [28], [29], [30] or molecules to graphene [31], [32], [33], [34]. Even though the FRET process as such has been studied for many years, modulation of the FRET processes is a relatively new research domain. Beyond the static control of FRET by varying the distance between donors and graphene [28, 35, 36], electrical control of FRET from individual QDs to graphene by electrical doping of graphene has been reported [15, 17, 29].

In a wider context, beyond graphene, external control of FRET by light [37], mechanical stimuli [38, 39], temperature and solvent [40], ions and pH [41], microfluidic flow [42], as well as electrical fields [43], has been achieved with the aim of constructing full color RGB displays [37], mechanical sensors [38], mechanical [39] and optoelectronic [14] switches, logic gates [41] or enhanced DNA sensors [42]. However, continuous modulation of the FRET efficiency between a donor and an acceptor by electric fields at room temperature has not been reported yet. As a typical FRET pair, fluorescein and R6G has been intensely studied in solutions [44, 45]. We report below the design and construction of a sandwich hybrid structure to tune the energy transfer efficiency between thin films of fluorescein and R6G molecules. In this structure, dye molecules were thermally evaporated in an ultrahigh vacuum device onto either side of freestanding SLG acting as donor and acceptor, respectively. We demonstrate that, by continuously doping the graphene via the applied bias voltage, we can modulate FRET between organic molecules located on opposite sides of the SLG.

## 2 Experimental sections

### 2.1 Graphene transfer process

SLG was grown on both sides of a Cu foil by chemical vapor deposition (CVD). Poly(methyl methacrylate) solution (commercial A4 PMMA solution) was spin-coated on top of the graphene/copper sample in order to generate a protective layer. The spin coating speed and time were 4000 rounds/min and 1 min, respectively. The spin-coated sample was kept in vacuum overnight and O_2_ plasma was applied to remove backside graphene. After that, an ammonium persulfate (APS) aqueous solution (0.1 g/mL) bath was applied to etch the copper foil. The PMMA/graphene/copper sheet was placed in an APS solution for 2 h to dissolve the metal and then the remaining PMMA/graphene film was transferred to a DI water bath to clean the graphene surface. At last PMMA/graphene was transferred onto the target substrates with the graphene in contact with the substrate and kept in a desiccator overnight. An acetone bath was used to remove the PMMA film. The fabrication process is illustrated in Figure S1.

### 2.2 Fabrication of a device for studies of electrical control of FRET between molecules (R6G/SLG/fluorescein)

To fabricate the device a holey silicon nitride (Si_3_N_4_) membrane with periodic hole arrays (Ted Pella, Inc.) was used as a substrate. The Si_3_N_4_ thickness is 200 nm, the diameter of the holes is 2.5 μm and the center to center hole spacing is 2.5 μm. In order to make the Si_3_N_4_ optically opaque, an Al film with 60 nm thickness was deposited on the backside of the membrane. CVD-grown SLG was transferred onto the silicon nitride membrane, as described above, and 7 nm Cr and 20 nm Au were deposited onto the edge of the SLG using a shadow mask, serving as electrodes for the device. Fluorescein and R6G molecular thin films with 10 nm thickness were deposited on either side of the SLG by thermal evaporation under a pressure of 1 × 10^−7^ mbar. The evaporation rates of molecules were monitored by a quartz balance. The final thickness of the deposited thin film was calibrated by means of atomic force microscopy (AFM). A single-crystal LaF_3_ substrate with (100) orientation and a thickness of 500 μm (MTI Corporation) was used as a solid-state electrolyte for gating. The excitation and emission spectra were obtained by using a Horiba/Jobin-Yvon Fluorolog-3 spectrofluorometer in air.

### 2.3 Measurement of PL intensity of the device as a function of the applied voltage

The PL of the device was excited employing a 405 nm laser (LDH-D-C-405 PicoQuant, Picoquant GmbH) operating in continuous mode and dispersed by an *f* = 500 mm spectrometer (Acton Research SP2500) equipped with a liquid nitrogen-cooled CCD (Princeton SPEX-10) and a low dark current hybrid photomultiplier (PMA 06, PicoQuant). The sample was placed in a vacuum recipient equipped with optical quartz windows in order to avoid photo-oxidation of the organic molecules. PL was excited from one side of the sample and was detected from the other side. The details of the set-up are shown in Figure S2. PL lifetimes were acquired using the same laser operating in pulsed mode (pulse width FWHM < 49 ps) at a repetition rate of 2.5 MHz. A Picoquant HydraHarp-400 time-correlated single photon counting (TCSPC) event timer with 1 ps time resolution was used to obtain the PL decays.

### 2.4 Raman measurements

Raman spectra were obtained using a Bruker SENTERRA II Raman Microscope equipped with a 532 nm laser and at 2 mW laser power.

## 3 Results and discussions

### 3.1 Electrical control of FRET between molecules via SLG

**Figure 1:**
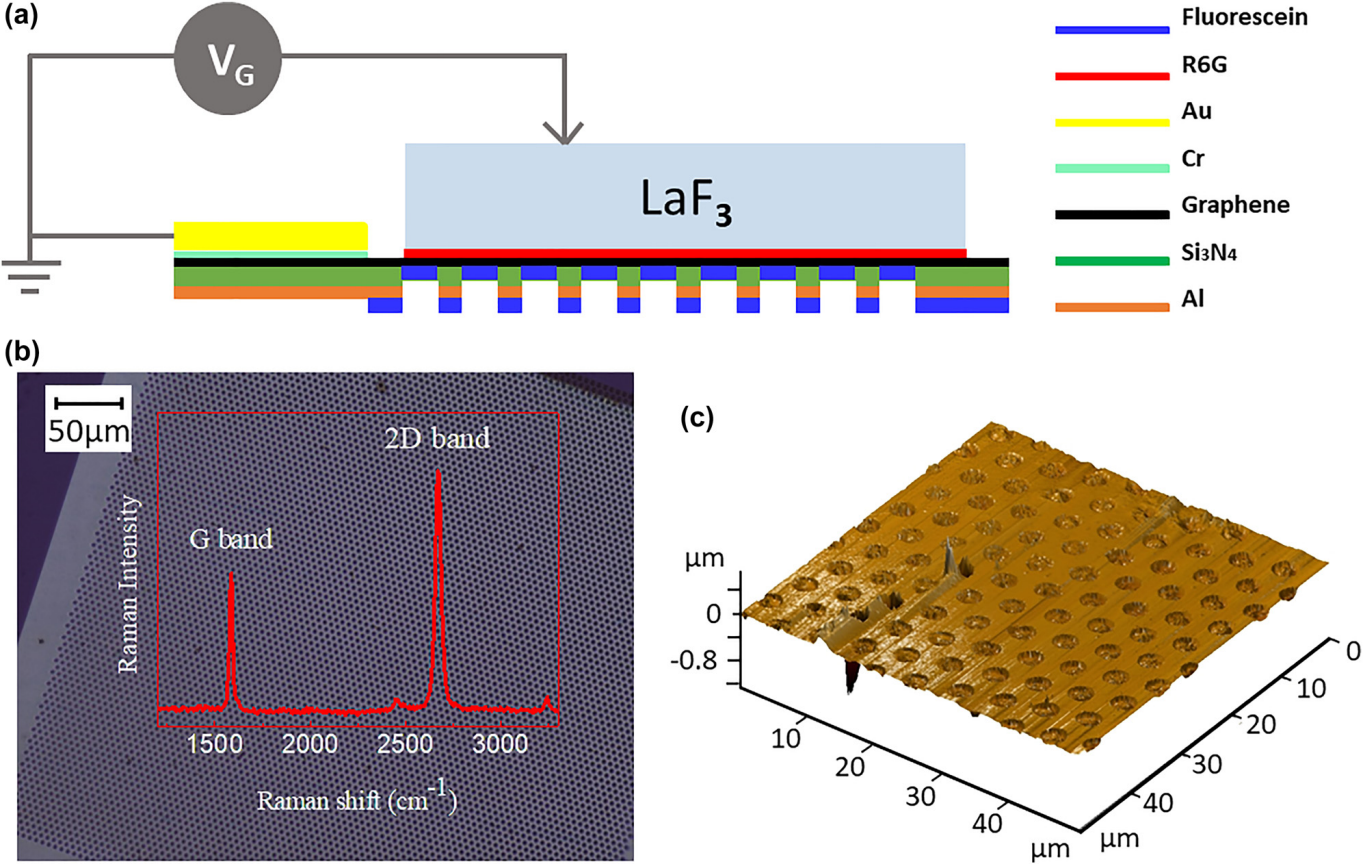
(a) Structure of the fabricated device. (b) Optical microscope image of SLG transferred onto the holey Si_3_N_4_ membrane. The insert image in (b) shows the Raman spectrum obtained from SLG on the holey Si_3_N_4_ membrane. (c) AFM image of SLG on the holey Si_3_N_4_ membrane, measured in dynamic mode.

To study the modulation of the FRET process between two molecules, a Sandwich R6G/SLG/fluorescein device has been designed and fabricated, using a holey Si_3_N_4_ membrane as the supporting substrate. The architecture of the device is illustrated in Figure 1(a). SLG was deposited onto the membrane which yielded free-standing SLG suspended over the micron-sized holes (diameter 2.5 μm). Because SLG is gapless and has an atomic thickness, it is applied here as an intermediate material to control the energy transfer process from donor to acceptor, or a donor to graphene by applying a bias voltage. The commercially available molecules fluorescein and R6G form a typical FRET pair and are therefore used here as a donor and acceptor. The molecules were thermally evaporated on either side of the SLG. The thickness of the molecular thin films was selected to be 10 nm as this guarantees a sufficiently strong PL signal while still allowing donor and acceptor molecules to interact [36, 46]. The contact electrodes were deposited on the SLG (5 nm Cr and 50 nm Au) using a shadow mask. The device was top-gated by a lanthanum trifluoride (LaF_3_) crystal, which is a solid-state electrolyte [47]. The fabrication details are described in the experimental section. Figure 1(b) represents an optical microscope image of SLG transferred onto the holey Si_3_N_4_ membrane substrates, demonstrating that graphene has been transferred successfully without significant damage or wrinkles. A three-dimensional representation of an AFM image of graphene suspended on the holey Si_3_N_4_ membrane is shown in Figure 1(c) for an area of 50 μm × 50 μm. The inset in Figure 1(b) exhibits the Raman spectrum of suspended graphene on the holey Si_3_N_4_ membrane, excited at 532 nm. The Raman spectrum provides conclusive evidence about the existence, defects and number of layers of graphene. The G peak at a Raman shift of ∼1580 cm^−1^ is due to the doubly degenerate zone center *E*_2g_ mode (in-plane optical mode) and the 2D band at a Raman shift of ∼2700 cm^−1^ corresponds to the second-order of zone-boundary phonons. By comparing the intensity ratio of *I*_2D_/*I*_G_ and considering the absence of the D peak which would be located at ∼1340 cm^−1^, it is confirmed that the graphene transferred to the holey membrane is SLG without significant defects [48, 49].

**Figure 2:**
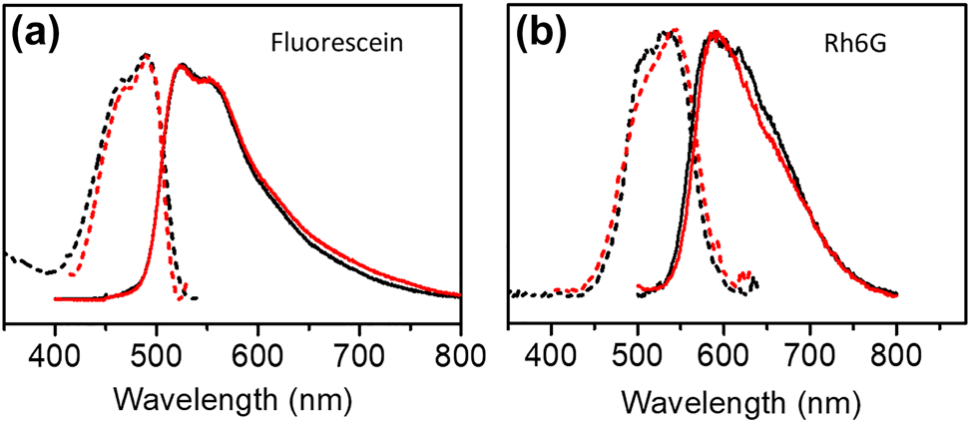
Excitation (dashed lines) and emission spectra (solid lines) of fluorescein (a) and R6G (b) deposited on SiO_2_ (black lines) and SLG/SiO_2_ (red lines), respectively, at the same thickness of the dye film (10 nm). Detection wavelengths for excitations spectra 560 nm/650 nm and excitation wavelengths for emission spectra 405 nm/430 nm for fluorescein/R6G, respectively.

In order to determine the optical properties of the fluorescein and R6G thin films, the corresponding excitation and emission spectra have been obtained. Figure 2 shows the normalized excitation and emission spectra of fluorescein and R6G thin films of 10 nm thickness deposited on top of SiO_2_/Si and SLG/SiO_2_/Si substrates. Figure 2(a) reveals a peak of the emission of fluorescein at ∼521 nm with a shoulder at ∼565 nm and a peak of the excitation spectrum at 488 nm with a shoulder at 465 nm. In Figure 2(b), the peak of the emission of R6G, on the other hand, occurs at ∼590 nm with a shoulder at ∼650 nm and the peak of the excitation spectrum a 540 nm accompanied by a shoulder at 509 nm. The emission range of fluorescein molecules that act as the donor is roughly from ∼500 nm to ∼600 nm which provides good overlap with the excitation range of R6G (acting as acceptor), approximately from 480 nm to 600 nm. SLG, on the other hand, is known to efficiently quench the emission of molecules deposited on top of it (compare also Figure S3), such that the emission intensity is expected to be lower than for a standard dielectric substrate.

**Figure 3:**
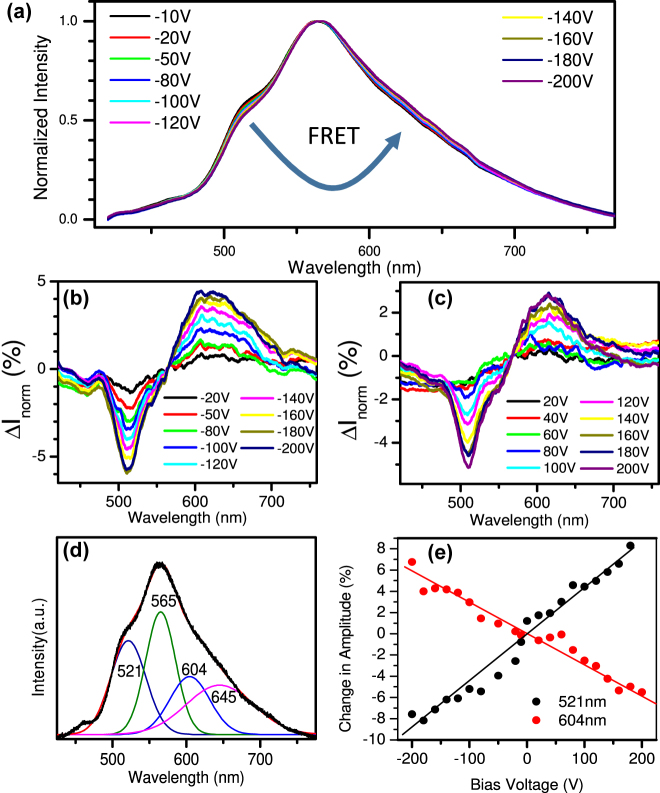
(a) Normalized PL spectra obtained from the device under negative bias voltages. (b) The changes in the normalized spectra obtained at negative bias voltages, as obtained by subtracting the normalized spectrum at a bias voltage of −10 V from the normalized measured spectra. (c) The changes in the normalized spectra obtained at positive bias voltages, as obtained by subtracting the normalized spectrum at a bias voltage of 0 V from the normalized measured spectra. (d) Fit of the PL spectrum at a bias voltage of 0 V to a sum of four Gaussian peaks located at 521 nm, 565 nm, 604 nm and 645 nm, respectively. (e) Relative intensity variation of Gaussian contributions at 521 nm and 604 nm, respectively, under different bias voltages obtained from fitting the absolute PL spectra as shown in Figure 3(d). The black and red solid lines are guidelines for the eye.

Figure 3(a) illustrates the normalized PL spectra obtained from the device under negative bias voltages. A transfer of intensity, monotonic in voltage for either sign of the voltage, from fluorescein deposited on one side of the SLG to R6G, deposited on the other side, is obvious. This inequivocally indicates voltage-controlled FRET between the molecules. For clarity Figure 3(b) and (c) shows the differential spectra. The maximum differences observed were ∼6% under a bias voltage of −200 V, and ∼5% under +200 V, respectively. The PL spectra were also fitted to a sum of Gaussian peaks located at 521 nm, 565 nm, 604 nm and 645 nm, respectively, as shown in Figure 3(d) for the spectrum obtained at zero bias voltage. Figure 3(e) shows the variation of the fitted amplitudes of the Gaussians centered at 521 nm and 604 nm, respectively, as a function of bias voltage. Again a decrease in intensity due to fluorescein and an increase of intensity due to R6G with increasing voltage, independent of the sign of the voltage is obvious. At the same time FRET between the molecules and SLG is reduced, as indicated by a monotonic increase of absolute peak PL intensity, as well as area under the emission spectrum, with increasing bias voltage (compare Figure S4).

## 4 Discussion

A very basic description of the FRET process is provided here in view of the following discussion. The donor, initially in the ground state, is transferred by the absorption of an external photon to an excited state. It transfers its energy to the acceptor by the nonradiative dipole–dipole energy transfer process, thereby being transferred back to the ground state. In such a nonradiative process, there are some conditions that should be satisfied which are: (i) The donor’s emission and acceptor’s absorption spectra should have a reasonable spectral overlap. (ii) the distance between donor and acceptor should be small, typically in the range of a few nanometers [26, 50], [51], [52]. In the case of donors and acceptors whose size is small compared to their distance, the dipolar approximation can be employed and the rate of dipolar resonance energy transfer is given by the following equation [46]:

(1) $$k_{\text{T}}=\frac{1}{\tau_{\text{D}}}\times {\left(\frac{R_{0}}{R}\right)}^{6}$$

with

(2) $$R_{0}^{6}=\frac{9000\;\text{ln}10\kappa^{2}\eta_{\text{D}}}{N_{\text{A}}128\pi^{5}n^{4}}\frac{\overset{\infty }{\underset{0}{\int }}\varepsilon_{\text{A}}\left(\tilde{\nu }\right)F_{\text{D}}\left(\tilde{\nu }\right){\tilde{\nu }}^{-4}d\tilde{\nu }}{\overset{\infty }{\underset{0}{\int }}F_{\text{D}}\left(\tilde{\nu }\right)d\tilde{\nu }}$$

Here *τ*_D_ is the fluorescence lifetime of the donor, *R* is the distance between donor and acceptor, and *R*_0_ is a distance parameter calculated from the spectroscopic and mutual dipole orientational parameters of donor and acceptor. In particular, *κ* represents the dipole orientation factor, *η*_D_ the quantum efficiency of the donor, *N*_A_ Avogadro’s number, *n* the refractive index of the medium and the integrals extend over the molar absorption coefficient $\varepsilon_{\text{A}}\left(\tilde{\nu }\right)$ of the acceptor and the fluorescence spectrum *F*_D_ of the donor. In the case of FRET to graphene, due to the extended nature of the electronic states, the dipolar approximation breaks down and Eq. (1) has to be modified. Swathi and Sebastian [53, 54], employing a noninteracting tight binding model for graphene derived a fourth-power distance dependence of FRET to graphene which was later confirmed experimentally for dye molecules as well as semiconductor quantum dots [28, 35].

Due to the particular band structure of SLG near the *K* and *K*′ points of the Brioullin zone it is expected that optical interband transitions in doped SLG can occur only at frequencies *ω* > 2*E*_F_, where the Fermi energy should be replaced by the chemical potential in the case of finite temperatures. This Pauli blocking naturally provides a mechanism for electrical control of FRET to SLG. Devices for the demonstration of electrical control of FRET to SLG, however, have been fabricated by the deposition of SLG on thermally oxidized SiO_2_/Si substrates. Electrical doping of SLG by back-gating such a device is limited to *E*_F_ corresponding to optical transition frequencies in the infrared. This can be readily estimated from the equation $\left|E_{\text{F}}\right|=\hbar v_{\text{F}}\sqrt{\pi N}$ and the charge density of SLG at a given bias voltage *e*N = CU/A = ɛɛ_0_U/d, where *ɛ* ≈ 4 is the dielectric constant of SiO_2_ and *d* ≈ 300 nm is the SiO_2_ thickness, resulting in *N* = 7.4 × 10^10^ cm^−2^·U/V. As the maximum voltage that can be applied to such a device is about 80 V in order to avoid electric breakdown of the SiO_2_ layer, the density of electrons or holes on the SLG is limited to *N* ≈ 5.9 × 10^12^ cm^−2^ and the Fermi energy to $\left|E_{\text{F}}^{\max}\right|\approx 0.28\;eV$ .

A mechanism different from Pauli blocking should arise from any other doping dependence of the optical properties, i.e., of the complex transverse and longitudinal conductivities, of SLG. In the case of the type of devices just discussed the relevant quantity is the real part of the conductivity, which corresponds to the absorption. In the case of the device shown in Figure 1, on the other hand, both, the real and imaginary parts of the conductivity will affect FRET between the donor and acceptor molecules. This is because FRET to graphene, determined by the real part, competes with FRET to the acceptor and because the imaginary part will also affect FRET from the donor to the acceptor, as can be concluded from the appearance of the factor *n* ^4^ in the denominator of Eq. (2) in the case of homogeneous environments.

Here it must be taken into account that FRET is a process that occurs in the near field of the donor, which requires considering a dependence of the conductivity on the wave vector and implies nonvertical optical transitions within SLG. Such transitions have been recently considered theoretically [55] and are actually also behind the work of references [53, 54]. It is obvious that in the case of the present device SLG is in the near field of both, donors and acceptors. The optical response of graphene is usually expressed as the response to an external vector potential in Fourier space. Therefore we apply Weyl’s theorem to establish the angular spectrum of the vector potential of the oscillating dipole (the emitting molecule) in the plane of the SLG (for details compare Section II of the SI):

(3) $$\begin{array}{ll} A\left(r\right) & =-i\omega \frac{\mu_{0}}{4\pi }p\frac{{\text{e}}^{i\mathrm{kr}}}{r}\\ & =\omega \frac{\mu_{0}}{8\pi^{2}}p\overset{\infty }{\underset{-\infty }{\iint }}{\text{e}}^{i\left(q_{x}x+q_{y}y+q_{z}\left|z_{0}\right|\right)}\frac{1}{q_{z}}dq_{x}dq_{y} \end{array}$$

Here, *z*_0_ represents the distance of the dipole to the SLG and the integral is over longitudinal as well as transverse plane waves in the plane of SLG. For the device used in the experiments a reasonable estimate for the *minimum* relevant distance is *z*_0,min_ = 0.5 nm, about half the size of a dye molecule. The integral in Eq. (3) can be split into contributions of propagating and evanescent waves and, by transforming the integral into cylindrical coordinates it is easy to see that the contribution of evanescent waves essentially extends from $q_{∥}=\sqrt{q_{x}^{2}+q_{y}^{2}}=\omega /c$ to *q*_∥_ ≈ 1/*z*_0_ (compare Section II of the SI).

**Figure 4:**
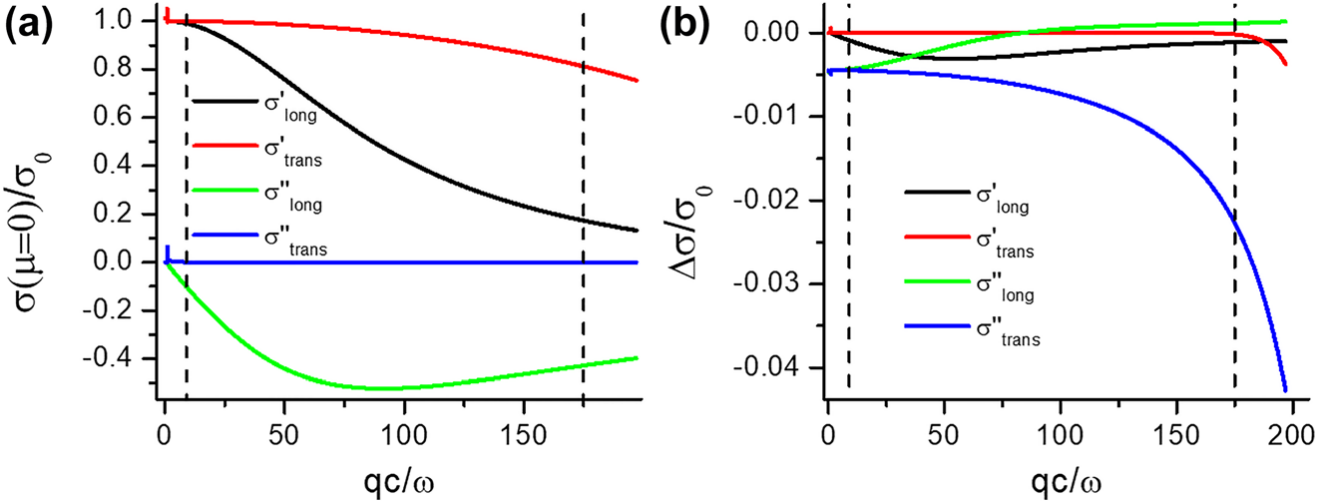
(a) Optical conductivities of graphene at zero bias and (b) doping dependence $\Delta \sigma =\sigma \left(\mu =0.3\;eV\right)-\sigma \left(\mu =0\right)$ as a function of wave vector, both in the propagating (*qc*/*ω* < 1) and the evanescent range (*qc*/*ω* > 1). *λ* = 550 nm, *T* = 300 K, *σ*_0_ = *e* ^2^/4*ℏ*. The dashed lines indicate *q* = 1/*z*_0_, *z*_0_ = 0.5 nm, marking the maximum evanescent wave vectors involved in the device employed for the experiment. In (b), the values of Δ*σ*/*σ*_0_ for $\sigma_{\text{long}}^{\prime }$ , $\sigma_{\text{trans}}^{\prime }$ in the propagating range are of the order of 10^−7^.

The doping-dependent longitudinal optical conductivity or, equivalently, susceptibility of graphene was investigated by Wunsch et al. [56] for the case *T* = 0 and at finite temperature by Ramezanali et al. [57], mainly motivated by the existence of longitudinal plasmons at low frequencies *ℏω* ≈ *μ*, *q* ≈ *k*_*F*_. The doping-dependent transverse optical conductivity of graphene, on the other hand, was studied by Mikhailov and Ziegler [58]. Gutiérrez et al. [59] have provided universal expressions for both, the transverse and longitudinal conductivity of graphene. All those approaches were based on the random phase approximation (RPA) which corresponds to the first order perturbation theory of the electron–electron interaction. Figure 4 illustrates how, within the RPA, the longitudinal and transverse optical conductivities of graphene at zero bias and its variation with the doping, defined here as the difference of the optical conductivities at a bias of 0.3 eV and at 0 eV, respectively, vary with the wave vector. The dashed lines indicate the maximum evanescent wave vectors involved in the device. The figures were calculated using the formalism of reference [59]. Additional information can be found in Section III of the SI.

Figure 4(a) demonstrates that, in the far evanescent range of wave vectors, the real parts of the optical conductivities are predicted by the RPA to deviate significantly from its “universal” value *σ*_0_. The largest variation with doping of *σ*′ is predicted for $\sigma_{\text{long}}^{\prime }$ , about 0.4% at *qc*/*ω* ≈ 50 (see Figure 4(b)). It is obvious that this dependence, being very small (≈10^−7^) in the range of propagating waves, is significant only in the range of evanescent waves. The values of the real parts are apparently still too small to explain the results obtained experimentally. The variation, however, is predicted to be negative, which results in a reduced probability for FRET to graphene at finite bias, in accordance with the observation. The variations with doping of the imaginary parts of the optical conductivities, in particular the one of $\sigma_{\text{trans}}^{\prime \prime }$ , on the other hand, are larger than those of the real parts and reach about 2.2% in this case at *q*_max_ = 1/*z*_0, min_. Due to the strong dependence of FRET on the permittivity of the intervening medium (compare the factor *n* ^4^ in Eq. (2)) the corresponding variation in the FRET efficiency is expected to be even larger. Figures S5 and S6 of the SI display the dependence of the optical conductivities of graphene, as predicted by the RPA, on doping and wavelength for the limiting wave vectors corresponding to *z* = 0.5 nm and *z* = 10 nm, respectively, i.e., for the minimum and maximum distance of molecules in the layers from the graphene sheet. A detailed understanding of what this variation of $\sigma_{\text{trans}}^{\prime \prime }$ means for FRET between molecules in the device shown in Figure 1, however, requires a microscopic model of FRET in the presence of the graphene sheet between the molecules, an issue beyond the scope of the present work.

The predictions of the RPA for the nonlocal response of the graphene sheet as a function of doping still appear somewhat smaller than required to explain the experimentally observed variation in FRET (Figure 3). Nevertheless, the graph does illustrate, once again, the essential non-locality of nano-optics, here in the special case of graphene. For another example see [60]. The reasons for the discrepancy will now be discussed.

Whereas early transmission experiments on graphene [61] in the visible range with limited precision seemed to confirm a universal and constant optical conductivity of graphene, it has been shown later theoretically [62], as well as experimentally [62], [63], [64], [65], [66], [67], [68] that, in the visible range, the optical properties of graphene are dominated by the van Hove singularity at the M-point of the Brillouin zone producing a very asymmetric peak (Fano resonance) in the optical conductivity of graphene, which extends far into the visible range, because excitonic effects shift this peak down to about 4.6 eV and the exciton binding energy was stated to be about 0.6 eV. The random phase approximation is not adequate to describe such excitonic effects. The GW–Bethe–Salpeter formalism [69] was therefore employed in the theoretical description.

Relevant for the experiments described above, on the other hand, is the doping dependence of its optical properties in the visible range, an issue that, due to experimental difficulties, has not been studied yet to great detail and with high accuracy. Mak et al. [70] employed transparent electrolyte top gates to achieve high doping levels in graphene and studied the corresponding changes in absorption, as spectroscopic ellipsometry was not possible due to the top gate. Within the limited accuracy of this approach of a few percent they mainly observed doping-related changes of the absorption in the spectral region of the exciton peak. Chang et al. [67], on the other hand, studied the variation of the complex optical conductivity of graphene with chemical doping, exposing graphene to nitric acid vapor. They reported changes mainly in the infrared part of the spectrum. Both experimental studies cited, however, employed spectroscopy in the far field, i.e., at *q* ≈ 0, whereas FRET, as discussed above, is essentially a near field effect. An experimental study of the doping-dependence of the *nonlocal* optical conductivity of graphene in the visible range of frequencies, i.e., in the range of the exciton-shifted van Hove/Fano resonance, has still not been reported, to the best of our knowledge. Motivated by the effects that the RPA produces in the evanescent range of *q* vectors we propose nevertheless that the electrical control of FRET reported in the present study is enabled in this way.

In view of further experimental efforts to control FRET between a donor and an acceptor on either side of a 2D material, it should be kept in mind that, in the case of MoS_2_/graphene van der Waals heterostructures, a strong doping dependence of the PL related to the MoS_2_ excitons was reported [71]. Whereas optical conductivities were not discussed these effects might enable efficient electrical control of FRET between molecules located on opposite sides of the heterostructure. The theoretical description of these effects, however, is significantly more complicated due to the necessity of including dynamic screening effects in the GW-BSE approach [72].

## 5 Conclusions

In conclusion, we have prepared a device incorporating a Sandwich structure, where we have transferred SLG to a holey Si_3_N_4_ membrane. Donor and acceptor molecules were then thermally evaporated in ultrahigh vaccum on either side of the free-standing SLG suspended over the micro-sized holes of the membrane. Au electrodes deposited on the SLG and a lanthanum trifluoride crystal in contact with the SLG allowed to continuously tune the energy transfer processes. According to our results, we successfully modulated the PL intensities by applying an external bias voltage. The experimental results revealed an approximate 6% modulation of PL intensity. By shifting the graphene’s Fermi level via electrical doping, the efficiency of FRET can be continuously modulated. To our knowledge this is the first time that an electrical modulation of FRET between donors and acceptors across SLG has been reported, making use of the atomic thickness of SLG. It should be pointed out that this is not of pure academic interest, as, for example, electrical control of FRET across SLG in principle provides access to highly sensitive detection of molecules using lock-in techniques and without the necessity to incorporate the donor (or acceptor) into the sample. In this context, operation of the device in a situation where either the donor or the acceptor resides in a liquid environment would be highly relevant for biological applications. This, however, will require considerable additional effort and exceeds the scope of the present work. We have also discussed the cause of the observed electrical effects on FRET and ascribe them to the doping dependence of the nonlocal optical conductivities of SLG in the range of evanescent wave vectors.

## Supplementary Material

Supplementary Material Details

## Notes

**Abbreviations:** FRET, Förster-type resonant energy transfer; QDs, quantum dots; rhodamine 6G; SLG, Single-layer graphene.
