# Supplementary material for: Electrical control of Förster resonant energy transfer across single-layer graphene
Source: Nanophotonics. 2022 Jun 10;11(14):3247–56. doi: 10.1515/nanoph-2021-0778 (PMC11501929; doi:10.1515/nanoph-2021-0778)
Supplement: Supplementary file 1 — Supplementary Material Details [file j_nanoph-2021-0778_suppl.pdf]

Electrical Control of Förster Resonant Energy Transfer Across Single-Layer Graphene

Yansheng Liu,[1,2,3] Miguel Angel Niño Ortí[1,4], Feng Luo[1,5] and Reinhold Wannemacher[1]**

[1] IMDEA Nanoscience, calle Faraday 9, Ciudad Universitaria de Cantoblanco, 28049, Madrid, Spain

[2] School of Science, Universidad Autónoma de Madrid, Ciudad Universitaria de Cantoblanco, 28049, Madrid, Spain

[3]Guangxi University of Science and Technology, School of Electrical and Information Engineering, 268 Donghuan Avenue, 545006 Liuzhou City, Guangxi Province, People´s Republic of China

[4]current address: Alba Synchrotron Light Facility, Cerdañola del Vallés, 08290 Barcelona,

[5]current address: School of Material Science and Engineering, Nankai University, 38, Tongyan Road, 300350 Tianjin, People´s Republic of China

Table of Contents

Page

Figure S1. Transfer of CVD-grown SLG onto holey Si_3N_4 membranes. 3

Figure S2. Set-up for measuring PL spectra and PL lifetimes. 4

Figure S3. PL decay of fluorescein and R6G thin films on SLG 5

Figure S4. Variation of absolute PL intensity emitted from the device 5

Supporting Discussion

Section I: Near field versus far field of an oscillating point dipole. 6

Section II: Range of Evanescent Wave Vectors for a Dipole Oscillating above the Graphene Sheet 6

Section III: Doping Dependence of the Transverse and Longitudinal Optical Conductivity of Graphene in the Random Phase Approximation 8

Figure S5. Longitudinal and transverse optical conductivities of graphene as a function of the chemical potential and the wavelength. 8

Figure S6.
Doping dependence of the optical conductivities of graphene at the limiting wave vectors for z = 10 nm and z = 0.5 nm in the evanescent range 9

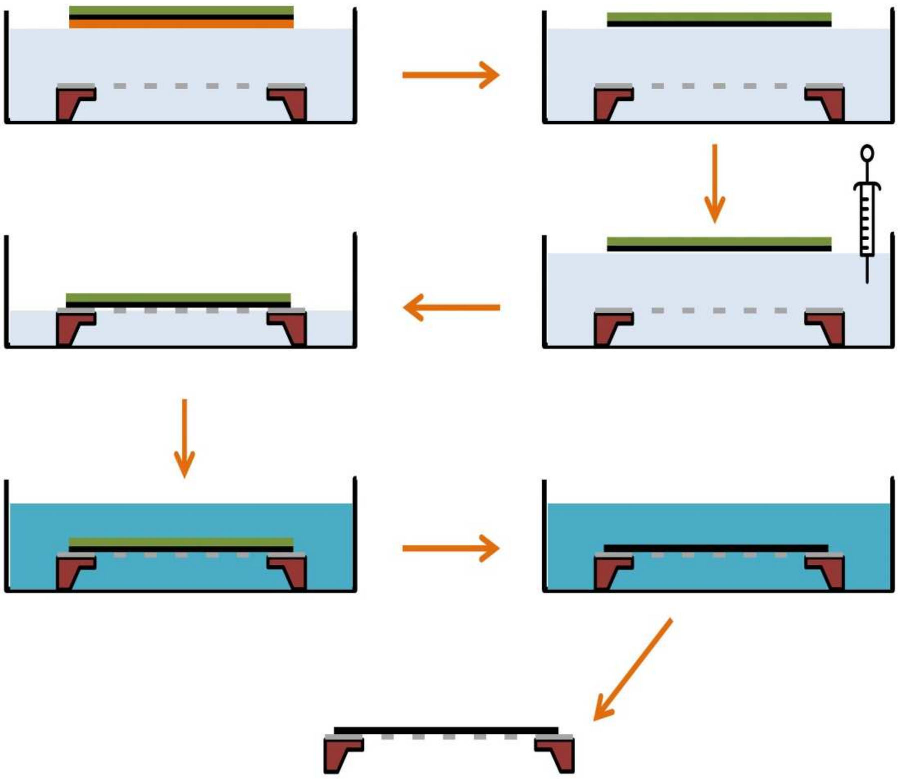

Figure S1. Transfer of CVD-grown SLG onto holey Si_3N_4 membranes.

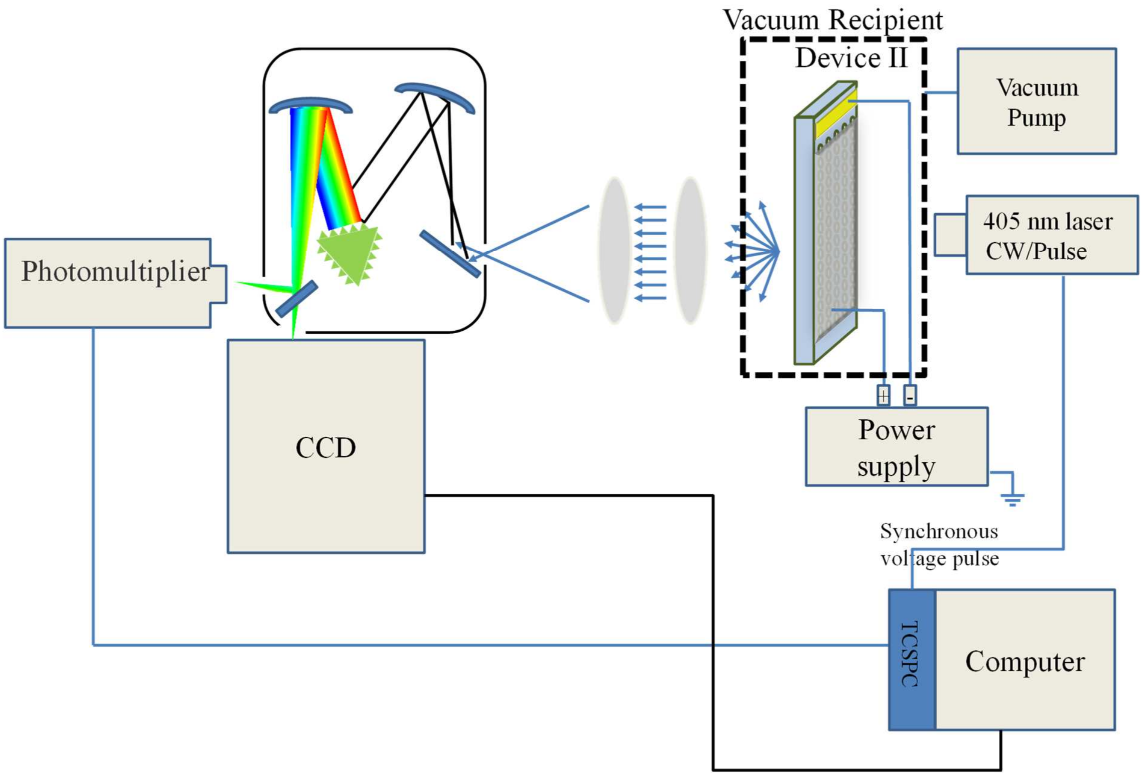

Figure S2. Set-up for measuring PL spectra and PL lifetimes.

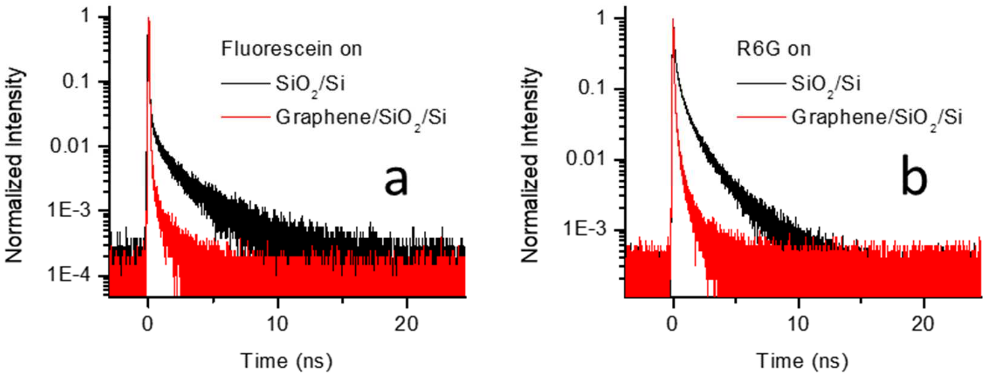

Figure S3. (a) PL decay of fluorescein thin film vacuum-deposited on SiO_2/Si and SLG/SiO_2/Si surfaces, respectively. Detection wavelength 525 nm (b) PL decay of R6G thin film deposited on SiO_2/Si and graphene/SiO_2/Si surface, respectively. Detection wavelength 596 nm.

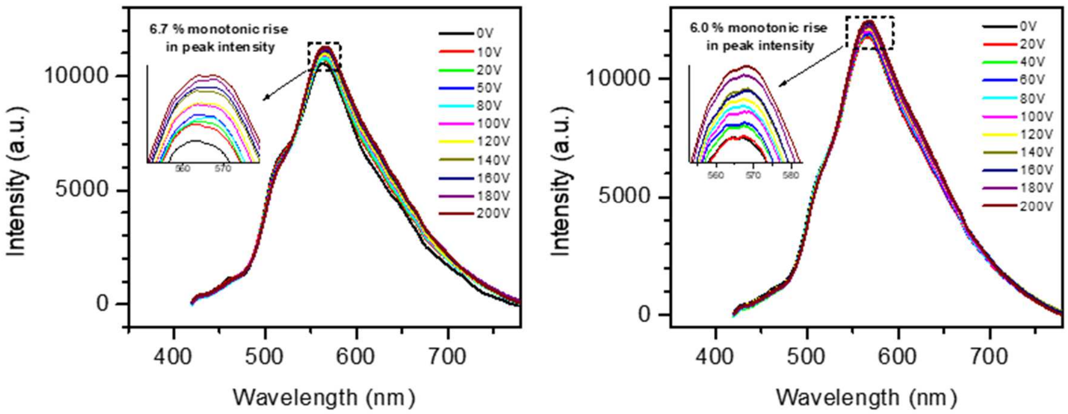

Figure S4. Variation of absolute PL intensity emitted from the device under (a) negative and (b) positive bias voltages.

Supporting Discussion

Section I: Near field versus far field of an oscillating point dipole

The electric field of an oscillating point dipole placed at the origin of the coordinate system may be written as [1]

$$\boldsymbol{E}(\vec{r}) = \frac{e^{ikr}}{4\pi\varepsilon_r\varepsilon_0} k^3 \left(\frac{1}{kr}\boldsymbol{\xi} + \frac{1}{(kr)^3}\boldsymbol{\zeta} - \frac{i}{(kr)^2}\boldsymbol{\zeta}\right) \quad (1)$$

with $\boldsymbol{\xi} = (\boldsymbol{e}_r \times \boldsymbol{p}) \times \boldsymbol{e}_r$, $\boldsymbol{\zeta} = \boldsymbol{3e}_r(\boldsymbol{e}_r \cdot \boldsymbol{p}) - \boldsymbol{p}$, $\boldsymbol{e}_r$ the unit vector in the direction of $\boldsymbol{r}$, $\varepsilon_0, \varepsilon_r$ the permittivity of vacuum and the relative permittivity of the medium, respectively, and $k = 2\pi/\lambda = \frac{\omega}{c}n$, λ the wavelength in the medium and n the refractive index of the medium. The first term represents the far field, the second the near field and the third the intermediate field of the dipole. The near field is identical to that of a static electric dipole. In the case of the fabricated device the graphene layer is clearly in the near field of the two molecules taking part in the FRET process.

Section II: Range of Evanescent Wave Vectors generated by a Dipole Oscillating above the Graphene Sheet

As the optical response of graphene is conveniently expressed as the response to an external vector potential in Fourier space it is useful to consider the latter for the case of a point dipole. In Lorenz gauge the vector potential fulfills the Helmholtz equation with the current density as the source:

$$(\nabla^2 + k^2)\boldsymbol{A}(\vec{r}) = \mu_0\mu_r\boldsymbol{j}(\vec{r}) \quad (2)$$

Given the current density of a point dipole $\boldsymbol{j}(\vec{r}) = -i\omega\boldsymbol{p}\delta(\boldsymbol{r})$ this means that the solution of eq. (2) is essentially given by the scalar Green´s function of the Helmholtz equation:

$$\boldsymbol{A}(\boldsymbol{r}) = -i\omega\frac{\mu_0}{4\pi}\boldsymbol{p}\frac{e^{ikr}}{r} \quad (3)$$

where a nonmagnetic medium in the frequency range of interest is assumed.

To obtain the vector potential in the plane of the graphene sheet the angular spectrum of eq. (3) must be used which is given by Weyl's identity [2]

$$\frac{e^{ikr}}{r} = \frac{i}{2\pi}\iint_{-\infty}^{\infty} dq_x dq_y e^{i(q_x x + q_y y + q_z|z|)}\frac{1}{q_z} \quad (4)$$

with $q_z = \sqrt{k^2 - q_x^2 - q_y^2}$. This results in

$$\boldsymbol{A}(\boldsymbol{r}) = \omega \frac{\mu_0}{8\pi^2} \boldsymbol{p} \iint_{-\infty}^{\infty} e^{i(q_x x + q_y y + q_z|z|)} \frac{1}{q_z} dq_x dq_y \tag{5}$$

Neglecting any possible anisotropic polarizabilities a plane wave propagating parallel to the z direction, normal to the graphene sheet, and polarized in the x direction, will excite a horizontal dipole parallel to x in a quantum dot or molecule placed near the graphene sheet. Plane wave components of the vector potential parallel to $\vec{q}$ will constitute the longitudinal vector potential and plane wave components parallel to $\vec{q}$ the transverse one. For $\boldsymbol{q} = q_x \boldsymbol{e}_x$ $\boldsymbol{A}$ is therefore purely longitudinal and for $\boldsymbol{q} = q_y \boldsymbol{e}_y$ $\boldsymbol{A}$ is purely transverse. To either one of those cases corresponds a proper response function of the graphene, i.e. the longitudinal and transverse optical conductivity of graphene, respectively. We have neglected here the continuity conditions of the field components in order to focus on the range of q values which contribute to the integral of equation (5). It is convenient to convert eq. (5) into cylindrical coordinates:

$$\boldsymbol{A}(\boldsymbol{r}) = \omega \frac{\mu_0}{8\pi^2} \boldsymbol{p} \int_0^\infty dq_\parallel q_\parallel \frac{e^{iq_z|z|}}{q_z} \int_0^{2\pi} d\varphi \, e^{iq_\parallel r_\parallel \cos(\varphi - \varphi_q)}$$

$$= \omega \frac{\mu_0}{4\pi} \boldsymbol{p} \int_0^\infty dq_\parallel q_\parallel \frac{e^{iq_z|z|}}{q_z} J_0(q_\parallel r_\parallel) \tag{6}$$

where φ, φ_q are the azimuthal angles of the vectors (x, y) and (q_x, q_y), respectively, $r_\parallel = \sqrt{x^2 + y^2}$, $q_\parallel = \sqrt{q_x^2 + q_y^2}$ and J_0 is the Bessel function of order zero.

The two-dimensional Fourier transform of eq. (6) in cylindrical coordinates simply is

$$\boldsymbol{A}(q_\parallel) = \omega \frac{\mu_0}{4\pi} \boldsymbol{p} \frac{e^{iq_z|z|}}{q_z} = k \frac{\mu_0 c}{4\pi} \boldsymbol{p} \frac{e^{iq_z|z|}}{q_z} \tag{7}$$

For $k|z| \ll 1$ the exponential will start to fall off at $|iq_z| \approx 1/|z|$, i.e. $q_\parallel \approx \sqrt{|z|^{-2} + k^2} \approx 1/|z|$, where z is the distance of the dipole to the graphene sheet. This determines the range of evanescent wave vectors $q_\parallel$ of the field generated by the oscillating dipole in the plane of the graphene sheet.

Section III: Doping Dependence of the Transverse and Longitudinal Optical Conductivity of Graphene in the Random Phase Approximation

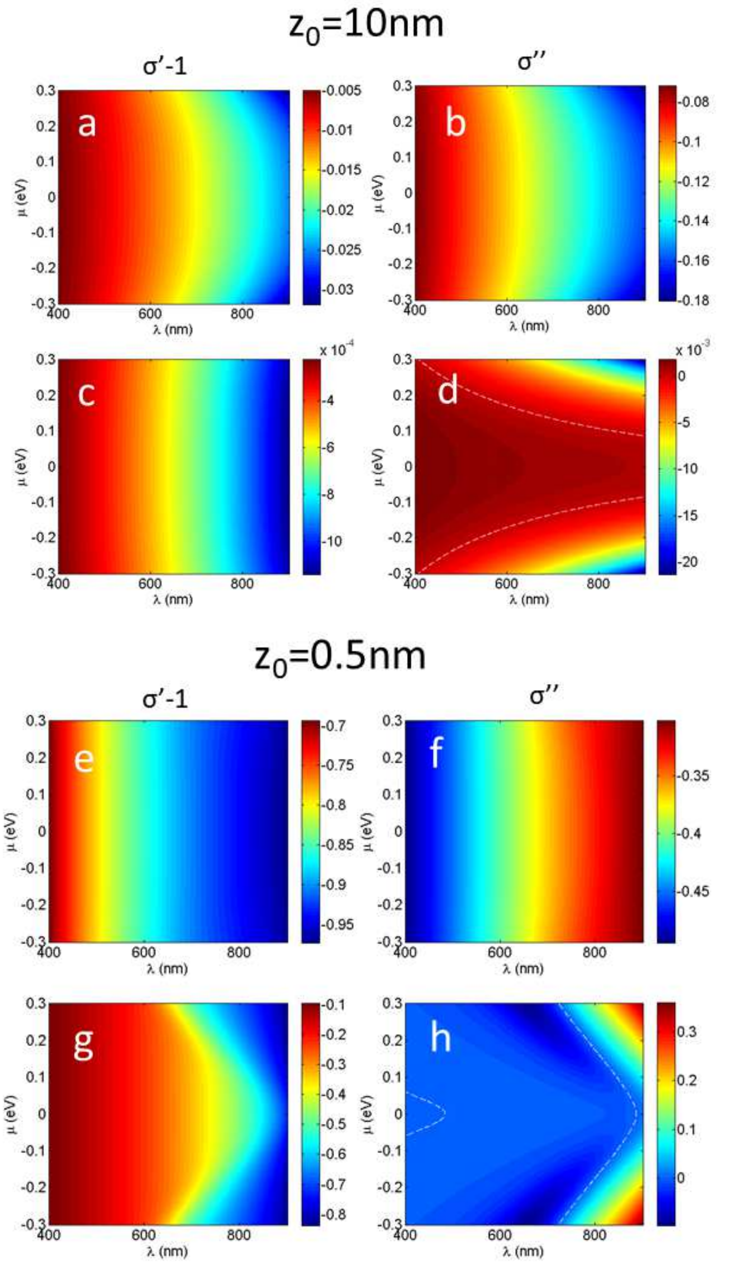

Figure S5: $Re(\sigma/\sigma_0) - 1$ (a,c,e,g) and $Im(\sigma/\sigma_0)$ (b,d,f,h) of the longitudinal (a,b,e,f) and transverse (c,d,g,h) optical conductivities of graphene as a function of wavelength λ in the visible range and of the chemical potential μ, as predicted within the random phase

approximation[1] for $T = 300K$ and $q_{\parallel} = 1/z_0$, $z_0 = 10nm, 0.5nm$, respectively. $\sigma_0 = e^2/4\hbar$. White dashed lines mark zero values.

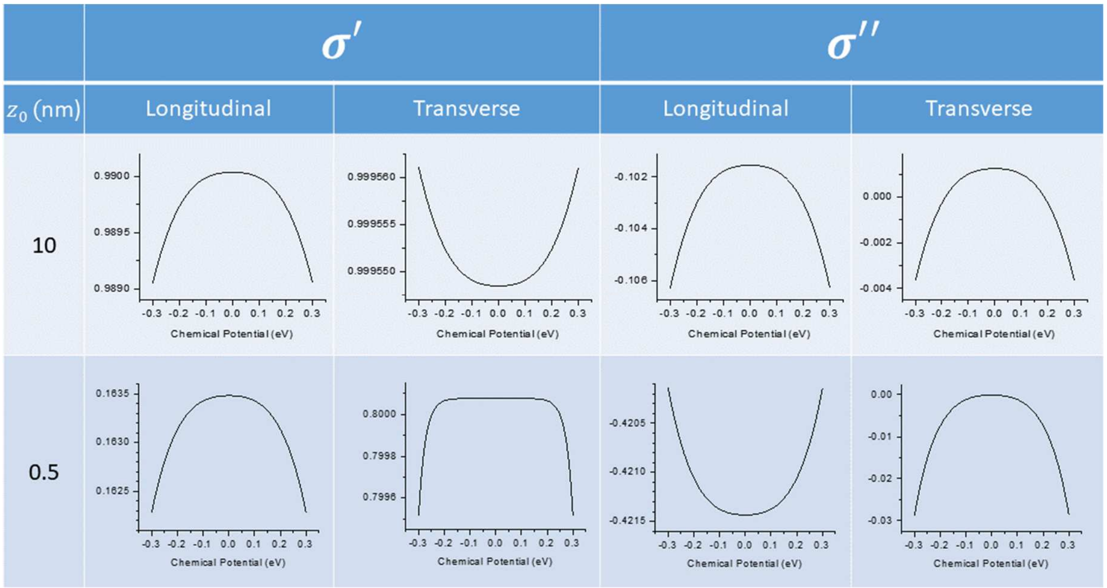

Figure S6:

Real and imaginary parts of the optical conductivities of graphene at T=300K as a function of the chemical potential, within the random phase approximation, at the wave vectors in the evanescent range, $q_{\parallel} = 1/z_0$, with $z_0 = 10nm$ and $z_{0,} = 0.5nm$, respectively.

References

[1] J. D. Jackson, Classical Electrodynamics, 3rd ed., Wiley, New York, eq. 9.18.

[2] H. Weyl,, Ann. Physik 1919, 365, 481-500.
